# Black Chokeberry (*Aronia melanocarpa*) Juice Supplementation Affects Age-Related Myocardial Remodeling in Rats

**DOI:** 10.3390/life15010023

**Published:** 2024-12-28

**Authors:** Elena Daskalova, Mina Pencheva, Slavi Delchev, Lyudmila Vladimirova-Kitova, Spas Kitov, Stoyan Markov, David Baruh, Petko Denev

**Affiliations:** 1 Department of Anatomy, Histology and Embryology, Medical Faculty, Medical University, 4000 Plovdiv, Bulgaria; slavi.delchev@mu-plovdiv.bg; 2 Department of Medical Physics and Biophysics, Faculty of Pharmacy, Medical University, 4000 Plovdiv, Bulgaria; 3 I-st Department of Internal Diseases, Cardiology Section Medical Faculty, Medical University, 4000 Plovdiv, Bulgaria; lyudmila.kitova@mu-plovdiv.bg (L.V.-K.); spas.kitov@mu-plovdiv.bg (S.K.); 4 Department of Otorhinolaryngology, Medical Faculty, Medical University, 4000 Plovdiv, Bulgaria; stoyan.markov@mu-plovdiv.bg; 5 Department of Software Engineering, Faculty of Mathematics and Informatics, Sofia University “St. Kliment Ohridski”, 1504 Sofia, Bulgaria; dbaruh@uni-sofia.bg; 6 Institute of Organic Chemistry with Centre of Phytochemistry, Bulgarian Academy of Sciences, Laboratory of Biologically Active Substances, 4000 Plovdiv, Bulgaria

**Keywords:** black chokeberry (*Aronia melanocarpa*), ageing heart, neovascularization, ACE2, CD34

## Abstract

Background: Cardiac aging is associated with myocardial remodeling and reduced angiogenesis. Counteracting these changes with natural products is a preventive strategy with great potential. The aim of this study was to evaluate the effect of *Aronia melanocarpa* fruit juice (AMJ) supplementation on age-related myocardial remodeling in aged rat hearts. Methods: Healthy male Wistar rats (n = 24) were divided into three groups: (1) young controls (CY)—age 2 months; (2) old controls (CO)—age 27 months; (3) AMJ group—27-month-old animals, supplemented with *Aronia melanocarpa* juice (AMJ) at a dose of 10 mL∙kg^−1^ for 105 days. After this period, the hearts of the animals were fixed, embedded in paraffin, and immunohistochemical and morphometric analyses were performed. Results: A higher vascular and capillary density was found in the hearts of the AMJ group, as compared to CO. The mean number of CD34+ cells in the myocardium increased by 18.6% in the AMJ group, as compared to CO (*p* < 0.05). Furthermore, the angiotensin converting enzyme 2 (ACE2) immunoexpression in the myocardium increased by 37% (*p* < 0.05) and the Proto-oncogene Mas receptor (MAS1) immunoexpression increased by 6% (*p* < 0.05) in the AMJ group, as compared to CO. Conclusions: As a result of the application of AMJ, noticeable neovascularization was found, which indicates improved myocardial nourishment. The present study demonstrates for the first time that polyphenol-rich AMJ can positively influence age-related microvascular myocardial remodeling in rats, thus outlining its potential as a preventive agent for healthy cardiac aging.

## 1. Introduction

With ageing, various alterations are observed in the myocardium, such as reduced blood perfusion, reduced formation of new vessels, and impaired vasodilation, which result in deterioration of its functional capacity [1]. One of the key factors in cardiovascular physiology and pathophysiology is the renin–angiotensin–aldosteron system (RAAS). The substance producing major effects in it is angiotensin II (Ang II), with vasoconstrictive, proinflammatory, and profibrotic properties [2]. The angiotensin converting enzyme 2 (ACE2) has been found recently to belong to RAAS. ACE2 is an integral membrane protein and functions as carboxypeptidase. The central role of ACE2 is to counteract ACE activity by reducing the bioavailability of Ang II and increasing Ang(1-7) formation. There is increasing evidence that ACE2 exhibits protective vascular effects by counteracting the deleterious effects of Ang II [3,4]. The presence of ACE2 is directly related to endothelial function and vascular tone. In the process of ageing, its expression is reduced [5]. Apart from that, it has been found that capillary density and angiogenesis in the heart, brain, and all organs of the body decrease with age, which ultimately results in physical and cognitive functional deficits [1,6].

The effects of ACE2 on neovascularization capacity have also been studied in vivo and in vitro. It has been experimentally found that overexpression of ACE2 promotes endothelial cell migration and results in the formation of new capillaries in tissues [7]. According to Singh et al., the RAAS system modulates the vasoreparative functions of CD34 cells. The ACE2/Ang(1-7)/Mas axis activates their vasoreparative potential, in contrast to the ACE/Ang II/AT1 axis, which attenuates these functions. The MAS1 (Proto-oncogene Mas) gene encodes a G protein-coupled receptor belonging to the family of transmembrane proteins. It functions as a receptor for angiotensin 1-7 and plays a role in hypotension, smooth muscle relaxation, and protection of cardiac muscles as a part of the RAAS system [8]. According to Endtmann et al., Ang II reduces the number of endothelial progenitor cells (EPCs) and causes EPC dysfunction in vivo and in vitro. [9]. It has been recently shown that ACE2 can influence the function of EPCs and protect ageing endothelial cells against hypoxia/reoxygenation-induced injury through the miR-18a/Nox2/ROS pathway [10,11].

CD34 is a cell-surface antigen expressed in multiple stem/progenitor cells including hematopoietic stem cells and EPCs [8]. CD34 was found to be highly expressed in small capillaries and in tissues that support early vessel development [12,13]. The studies of Ribatti provided compelling evidence for a novel role of CD34+ cells in postnatal vasculogenesis [14]. For this reason, CD34+ cells are often defined as endothelial progenitor cells. It is now well documented that these cells, in response to hypoxia-regulated factors, such as stromal-derived factor 1 (SDF) and vascular endothelial growth factor (VEGF), proliferate and migrate to areas of ischemia and accelerate vascular repair, thereby preventing tissue damage [15]. Autologous cell therapy using a population of CD34+ cells has already emerged as a promising approach for the treatment of ischemic myocardial disease [16]. CD34+ cells recently have been used to improve therapeutic angiogenesis in various diseases and also have potential for therapeutic application in vascular, ischemic, and inflammatory pathological conditions [17].

The application of natural products for the purpose of improving the functional abilities of the myocardium in the elderly is a valuable prophylactic strategy. The fruits of the plant *Aronia melanocarpa* possess proven anti-inflammatory and antioxidant properties, that underlie their hepatoprotective, immunomodulating, antimutagenic, anticarcinogenic, lipid lowering, antidiabetic, and antihypertensive effects [18,19,20,21,22,23]. Recently, black chokeberry products and extracts have attracted serious scientific interest in regard to their antiaging properties and geroprotective activity. It was concluded that using different disease models, the antiaging effect of aronia extracts are expressed as lifespan extension, anti-proliferative activity, improvement of glucose and lipid metabolism, amelioration of neurodegenerative disorders, and gastroprotective effects, and the underling mechanism could be associated with the hormesis effect, activation of antioxidant defense, modulation of insulin/IGF-1 signaling, and anti-inflammatory activity [23]. In a model using healthy aged rats, we have revealed that black chokeberry fruit juice, rich in phenolic compounds and particularly anthocyanins, shows a neuroprotective effect by increasing the density of nerve fibers in the hippocampal perforant pathway [24] and modulates ACE2 immunoexpression and diminishes age-related remodeling of coronary arteries in old rats [25].

The effect of natural compounds on the morphology of myocardium is barely researched. Most of the studies investigate the beneficial effect of different plant extracts and preparation (rosemary, Nutmeg-5, black bean coat, etc.) on cardiac remodeling after myocardial infarction or in models of non-ischemic cardiomyopathy [26,27,28]. However, to our knowledge there is only one study investigating the effect of natural products (curcumin) on cardiac angiogenesis in a model of healthy aged rats. This study found that curcumin improves angiogenesis in the heart of aged rats via TSP1/NF-κB/VEGF-A signaling [29]. Based on these findings, we hypothesize that polyphenol-rich black chokeberry juice would have a beneficial effect on age-related vascular myocardial remodeling in rats, and this defined the purpose of our study.

## 2. Materials and Methods

### 2.1. Aronia melanocarpa Juice

*Aronia melanocarpa* juice (dry solids content 18.8 °Bx) was obtained as described in Daskalova et al. [24]. Briefly, five kilograms of frozen fruit were defrosted at room temperature and homogenized in a laboratory blender. The homogenate was transferred into a brown-glass bottle and incubated in a thermostatic shaking water bath at 60 °C for one hour. After that, the pulp was filtered through a cheesecloth, and the liquid fraction obtained was centrifuged and used for this study. The delivery of the black chokeberry berries was made by licensed farmer Todor Petkov (Kazanlak, Stara Zagora district, Bulgaria) in the stage of full maturity in August 2017. The detailed analysis protocols of the juice used in the current study and its chemical composition (Table 1) are described in Daskalova et al. [24].

### 2.2. Test Animals

The detailed animal protocol is given in Daskalova et al. [24]. Briefly, male Wistar rats (n = 24) were provided by the Vivarium of Medical University, Plovdiv, where they were maintained under standard laboratory conditions (housed in polypropylene cages in a controlled clean-air environment at a temperature of 22 ± 3 °C, a 12 h light/dark cycle, and a relative humidity of 60 ± 5%). The rats were divided into 3 groups: (1) young controls (CY)—aged 2 months without AMJ supplementation (n = 8); (2) old controls (CO)—aged 27 months without AMJ supplementation (n = 8); and (3) the AMJ group—27-month-old animals, supplemented orally with AMJ (10 mL∙kg^−1^), diluted 1:1 in their drinking water for 105 days (n = 8). The duration of supplementation was based on our previous studies (Daskalova et al., 2019). Rats were on a standard rodent chow (containing 13.45% protein, 51.6% carbohydrate, and 3.40% fat) and tap water ad libitum. The daily dose of juice was calculated for every animal after body weight measurement (twice a month). The animals from the AMJ group received clear water after ingesting the daily dose of diluted juice. For the whole experimental period, every animal consumed approx. 440 mL fruit juice. At the end of the experimental period, the animals were anesthetized with i.m. Ketamin at 90 mg/kg and Xilazine at 10 mg/kg (Ketamine hydrochloride, QN01AX03; BREMER PHARMA GmbH Production, 34414 Warburg, Germany; Xylazine, Bioveta Romania SRL, 400 089 Cluj-Napoca, Romania) and euthanized by cervical decapitation. After that, the hearts of the animals were fixed in 10% neutral formalin and embedded in paraffin, after which immunohistochemical, morphometric, and statistical analyses were performed.

### 2.3. Immunohistochemistry

For all the cases included in this study, immunostaining was performed through a fully automated and standardized procedure, using an Autostainer Link 48 (Dako, Agilent Technologies Inc., Glostrup, Denmark). Paraffin sections (5 µm-thick) were treated for 20 min with a EnVision FLEX Visualization Systems (Dako, Agilent Technologies Inc., Glostrup, Denmark). Endogenous peroxidase was blocked with 3% hydrogen peroxide for 5 min. Then, the sections were incubated for 30 min with the α-SMA monoclonal antibody 1:5000 (A-2547, Sigma Chemicals, St. Louis, MO, USA), ACE2 polyclonal antibody 1:200 (E-AB-12224, Elabscience Biotechnology Inc., Houston, TX, USA), MAS1 monoclonal antibody 1:300 (sc-390453, Santa Cruz Biotechnology Inc., Santa Cruz, CA, USA), and CD34 (QBEnd/10) primary antibody (05299233001, Roche, Rotkreuz, Switzerland).

The EnVision FLEX Visualization Systems containing 3,3-diamino-benzidine dihydrochloride and hematoxylin were used for visualization. Negative controls for the four receptors were run in parallel by omitting the primary antibody under the same conditions. Stained sections were permanently mounted with Canada balsam.

All microphotographs were taken using a Leica DM3000 microscope (Leica Microsystems, Wetzlar, Germany) combined with a Flexocam C3 digital camera (Leica Microsystems, Wetzlar, Germany).

### 2.4. Morphometric Analysis

The morphometric analysis involved tissue slices 5 µm in thickness, obtained from rat hearts immediately below the coronary sulcus. All studies were performed on the left ventricle. All measurements involved five slices per animal.

The intensity of the immune reaction in the myocardium of the left ventricle was measured on the slices immunostained for ACE2 and MAS 1. Using software DP–Soft ver. 3.2 (Olympus, Tokyo, Japan)., the average intensity of pixels was recorded in arbitrary units (AUs). 

To calculate the number of CD34+ cells, areas showing maximal vascularization were initially identified at ×100 magnification [1]. These areas were then photographed at ×200 magnification. A 500/500 pixel grid was overlaid on the microphotographs using the software, and the mean number of CD34 immunopositive cells and the mean number of cells per unit area (cell density) were measured based on the available cell nuclei. When counting along the quadrant boundary lines, only the nuclei with 2/3 of the cells entering the quadrant were counted.

The average number of blood vessels on the cross-section of the left ventricle and immunoexpression intensity for α-SMA and CD34 were estimated semiquantitatively on the basis of the immunohistochemical reaction in the heart wall using the following legend: −, absent; +, weak; ++, moderate; +++, strong expression. The measurements were performed using the DP–Soft ver. 3.2 software (Olympus, Tokyo, Japan).

### 2.5. Statistical Analysis

The results are presented with mean values and standard deviation (SDs). The mean values for the three groups were compared with the Tukey HSD or Games–Howell tests—One-Way ANOVA Post Hoc Tests—depending on equal variances being assumed or not assumed. The correlation between CD34+ cells and cell density is measured by Pearson’s coefficient (r). *p* < 0.05 was considered significant. Data was analyzed using IBM SPSS Statistics v.25 software products.

## 3. Results

### 3.1. α-SMA Immunoexpression and CD34 Immunoexpression

**Figure 1:**
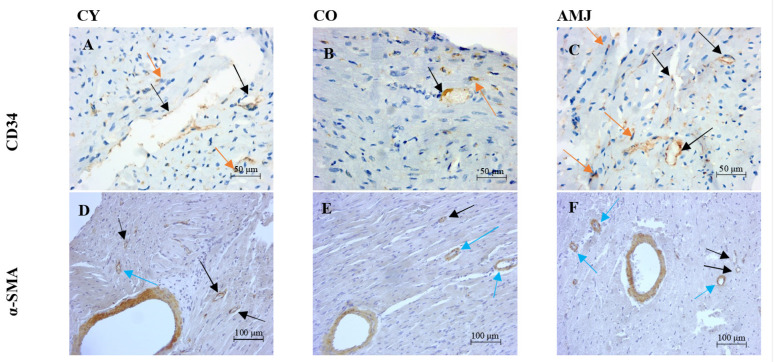
Rat myocardium. (**A**–**C**): CD34 immunoexpression (magn. ×200, scale bar = 50 µm) in capillaries (black arrow) and CD34+ cells (orange arrow); (**D**–**F**): α-SMA immunoexpression (magn. ×100, scale bar = 100 µm) in capillaries (black arrow) and small-caliber blood vessels (blue arrow).

In the current study, we used immunohistochemical analysis for α-SMA and CD34 in the myocardium in the three experimental groups to examine the amount of blood vessels and the appearance of new ones in the heart wall. In the myocardium, alpha α-SMA immunopositive structures presented as stained brown (Figure 1D–F).

In the myocardium, we found α-SMA expression is particularly high in the walls of blood vessels of different calibers, including capillaries. In all experimental animals, distinct brown staining of the walls of blood vessels of different calibers was found. There were also different numbers of single cells positive for α-SMA, which indicates the presence of pericytes and myofibroblasts. In the group of aronia-supplemented old animals, we found a higher intensity of α-SMA immunoreaction in the walls of large vessels compared with that in the CO group. The greater density of thin-walled vessels and capillaries, as well as the presence of many scattered single cells in the myocardium, was also striking in the AMJ group compared with the CO group (Figure 1D–F, Table 1 and Table 2).

CD34 is a membrane protein that is a marker for vascular endothelial cells, vascular progenitor cells, fibroblasts, and muscle progenitor cells. We used CD34 as a marker for vasculogenesis. In the myocardium, CD34 immunopositive structures presented as stained brown (Figure 1A–C).

In all three groups, we observed distinct expressions of CD34 in the walls of thin-walled blood vessels and in capillaries, which allowed us to distinguish and quantify them. Semiquantitative analysis of the intensity of the CD34 immunoreaction in the myocardium showed that it was higher in the aronia-supplemented group compared with the CO, reaching the level of intensity in the CY (Table 2).

Semiquantitative analysis of the distribution of CD34+ cells in the myocardium showed that both total blood vessel density and capillary density in the myocardium were higher in the AMJ group compared with that in the old CO controls. As is evident from Table 3, the old controls had lower vascular density compared with the young controls, which is a manifestation of the natural age-related myocardial remodeling.

**Figure 2:**
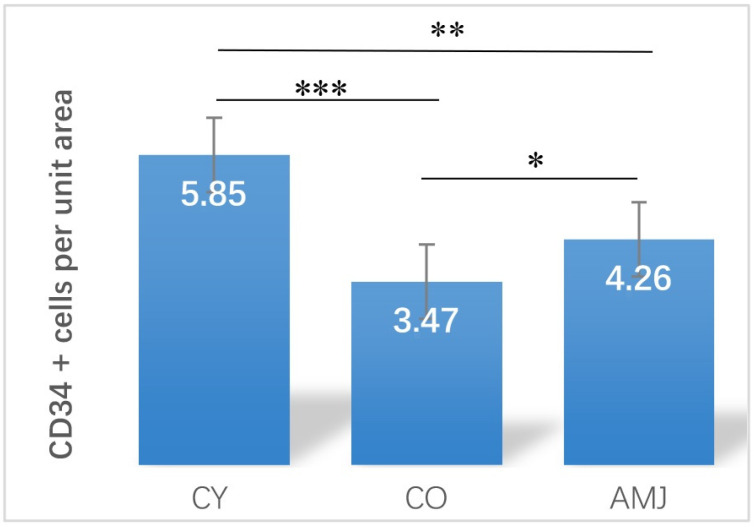
CD34 + cells per unit area. *** *p* < 0.001, ** *p* < 0.01, and * *p* < 0.05.

Statistical analysis of the mean number of CD34+ cells per unit area showed significantly lower values in CO versus CY (*p* < 0.01), a manifestation of the natural aging process. In the AMJ-supplemented group, the mean number of CD34+ cells were significantly higher compared to the CO (*p* < 0.05), which is an expression of their active proliferation (Figure 2).

**Figure 3:**
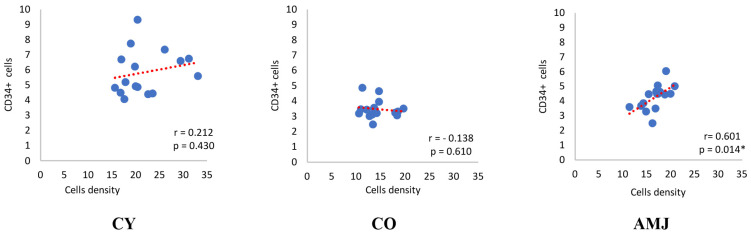
Correlation between CD34+ cells per unit area and cell density; r—Pearson Correlation; p—significance level, * *p* < 0.05.

The analysis of the data in relation to the number of CD34+ cells and the total number of cells per unit area reveals a weak positive correlation in the CY group (r = 0.212, *p* = 0.430) and a very weak negative correlation in the CO group (r= −0.138, *p* = 0.610). However, in both cases, the low values and their statistical insignificance reveal a disproportionate and non-random distribution of CD34+ cells (Figure 3). In the AMJ group, there was a high positive correlation between the number of CD34+ cells per unit area versus the mean number of cells per area (r = 0.601, *p* = 0.014) (Figure 3).

### 3.2. ACE2 Immunoexpression and MAS 1 Immunoexpression

**Figure 4:**
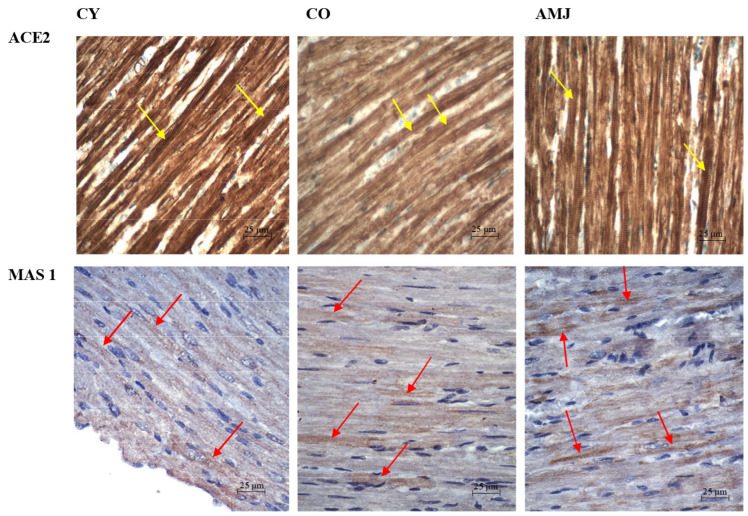
Rat myocardium. ACE2 immunoexpression in cardiomyocytes (yellow arrows), (magn. ×400, scale bar = 25 µm) and MAS 1 immunoexpression in cardiomyocytes (red arrows), (magn. ×400, scale bar = 25 µm).

Immunohistochemical staining in rat myocardial sections showed that ACE2 was widely expressed in cardiomyocytes, cardiac fibroblasts, and coronary endothelial cells (Figure 4). MAS1 protein was detected in cardiomyocytes as well as in coronary arteries and capillaries in all three experimental groups (Figure 4).

**Figure 5:**
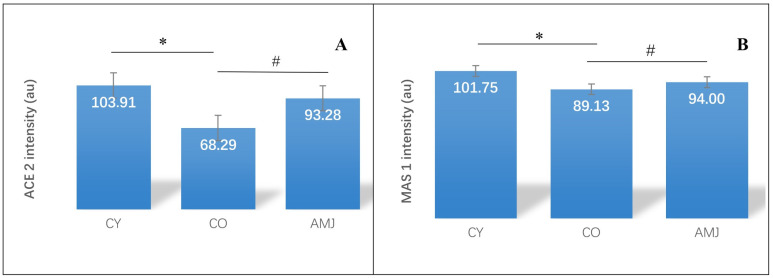
Rat myocardium. (**A**) ACE2 immunoexpression intensity: * *p* < 0.05; # *p* < 0.05; (**B**) MAS 1 immunoexpression intensity: * *p* < 0.05; # *p* < 0.05.

Morphometric analysis of immunoreaction intensity of ACE2 in myocardium showed significantly higher values in the AMJ group compared to the CO group (*p* < 0.05). Comparison between CY and CO showed a significantly higher intensity (*p* < 0.05) in the CY group (Figure 5). This confirms the decrease in enzyme activity that occurs with age (Figure 5). Morphometric analysis of MAS 1 immunoreaction intensity showed significantly higher values in the AMJ group compared to the CO group (*p* < 0.05). Comparison between CY and CO showed a significant difference (*p* < 0.05) in favor of CY.

**Figure 6:**
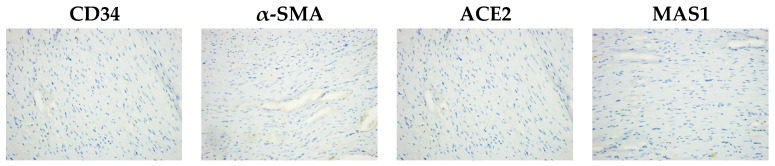
Negative controls of the CD34, α-SMA, ACE2, and MAS1 receptor by the omitting of primary antibodies in the tissue sample. The magnification for all images is ×200.

Figure 6 shows the micrographs of the negative controls of the four immunoreactions obtained by omitting the primary antibodies in the immunohistochemical protocol.

## 4. Discussion

Our results clearly demonstrate the formation of new blood vessels in the myocardium of old rats after the supplementation with *Aronia melanocarpa* fruit juice. This is a remarkable result, especially given the age-related remodeling which already had occurred in the myocardium.

In the aging heart, devoid of disease in certain instances, there is an identified thickening of the left ventricular myocardium. Conversely, in other cases where the thickness of the left ventricle remains unchanged, an enlargement of cardiomyocytes occurs alongside a reduction in their overall count. Simultaneously, there is an observed increase in the relative proportion of collagen fibers within the myocardial interstitium [30]. Several morphometric studies have shown that myocardial growth results primarily from an increase in contractile cell volume [31,32,33]. Cardiac myocytes in rats retain some capacity for proliferation until about weaning age [34]. Concomitant changes in the biochemical and electrophysiological properties of the aging myocardium, coupled with a reduced number of enlarged muscle cells in the ventricles, may provide an explanation for the limited cardiac function under conditions of increased workload in the aging heart [30]. Studies on the growing rat heart have shown that the concentration of capillaries in the left ventricular myocardium increase rapidly after birth, reaching its maximum concentration around the first month [35,36]. The continuous growth of the myocyte population in later stages of life results in an increase in myocyte diameter, which in return leads to a lateral displacement of neighboring capillaries, thereby decreasing the capillary density [35,36].

Reduced capillary concentration leads to a reduced endothelial surface area available for tissue oxygen exchange and a greater mean diffusion distance for oxygen transport to myocytes. These capillary characteristics may provide a structural basis for local ischemia that results in diffuse loss of single myocytes throughout the ventricular wall in older rats [1]. According to the experimental data of Iliev et al., capillary network development during the postnatal period in rats declines significantly and lags behind the increase in the surface area of cardiac muscle cells in both the left and right ventricle [1]. Vascular wall thickening with collagen deposition and increased stiffness of large coronary arteries have been found in old rats, which was shown in our previous research [25,37]. This further contributes to the deterioration of myocardial trophic factors during the aging process. Moreover, the increase in heart weight observed in aging humans and animals does not coincide with a proportional enlargement of the coronary tree, encompassing the capillary microvasculature. This lack of proportional expansion leads to relative oxygen deficiency, initiating dystrophic changes in myocardial tissues [37].

The existing literature contains limited data regarding the effects of black chokeberry fruits on the process of neovascularization of the heart [38]. The process of forming new blood vessels is associated with the activity of EPCs and mature endothelial cells. Angiogenesis is a crucial component of tissue remodeling and regeneration of ischemic tissues [6,39]. Our results show a significantly higher mean number of CD34+ cells in the myocardium of the AMJ group in comparison with the CO group and a positive correlation between the number of CD34+ cells and the cell density in the myocardium, which may be due to active proliferation of EPCs or their migration from the blood. Stimulation of EPCs in the myocardium may be associated with increased ACE2 and MAS activity as a result of supplementation.

Given the variability in the distribution of CD34+ cells, it is plausible that other factors, such as inflammatory responses and general cell proliferation, could influence these results. Evidence from various studies highlights the dual role of CD34+ cells in inflammation and tissue repair. For instance, CD34+ cells have been shown to migrate to inflamed areas in response to chemotactic signals like SDF-1α, which is upregulated in inflammatory conditions [40]. This suggests that inflammation may affect the mobilization, homing, and function of CD34+ cells. Additionally, CD34+ cells demonstrate anti-inflammatory effects through the secretion of IL-10 and the suppression of pro-inflammatory cytokines such as TNF-α and IL-6, which could modulate the systemic inflammatory milieu [41,42]. Furthermore, CD34+ cells play a role in angiogenesis and vascular repair by responding to hypoxia-regulated factors such as VEGF, potentially linking their distribution to ischemic conditions rather than just baseline proliferation [43].

Given these findings, the variability in CD34+ cell levels could reflect a dynamic interplay between inflammatory responses, vascular repair mechanisms, and underlying systemic conditions, including age-related vascular remodeling or myocardial ischemia [44]. These considerations suggest that both inflammation and proliferation are likely contributors to CD34+ cell variability, warranting further investigation into how these factors interact and influence their therapeutic potential.

The data from preclinical studies suggest that CD34+ cells differentiate into endothelial cells, integrate into the vasculature, and secrete angiogenic factors, promoting vessel regeneration in the microcirculation and improving myocardial perfusion in ischemia-induced tissue injury [17]. Migration to areas of ischemia is an important property of CD34+ cells that determines their reparative function. This function is mainly modulated by hypoxia-regulated factors such as VEGF and SDF [45].

In our study, we identified a correlation between the significantly increased activity of the ACE2/MAS1 axis and the increased number of CD34+ cells, as well as an increased amount of myocardial micro vessels after aronia juice intake, which indicates their interconnection. Singh’s study showed that activation of ACE2 or the Mas receptor induced a migration of CD34+ cells that was comparable to the response elicited by SDF. The effects of Ang-(1-7) on migration are mediated by Mas receptor activation [8].

Several studies have demonstrated that ACE2 is highly expressed in vascular endothelial cells and is associated with improved endothelial cell function [46,47,48]. The effects of ACE2 on the neovascularization capacity have also been studied in vivo and in vitro. Experimental findings indicate that overexpression of ACE2 promotes endothelial cell migration and results in the formation of new capillaries in the tissues [7]. Therefore, it can be inferred that ACE2 operates within the endothelium to modulate Ang II levels by facilitating its breakdown and augmenting the generation of Ang (1-7), a molecule endowed with vascular-protective properties. Improvement of endothelial function is the likely mechanism through which ACE2 exerts its cardio-vascular protective effect [7]. It was found that Ang (1-7) increases the vascular reparative function of CD34+ cells isolated from mice with diabetes [49]. It is very likely that the increased activity of the ACE2/MAS1 axis is a cause of the increased number of CD34+ cells and the corresponding increase in the number of micro vessels in the myocardium of old rats in our experiment. The RAAS also plays a key role in modulating the function of EC by regulating the production of NO, reactive oxygen species, and factors of inflammation [50,51,52].

The antioxidant and anti-inflammatory attributes of aronia constitute an additional mechanism for modulating RAAS activity, contributing to cardioprotective effects. We hypothesized that increased ACE2/MAS1 expression could result from systemic changes and not only from local myocardial remodeling. Supporting evidence from studies on aronia suggests that it may affect the cardiovascular system systemically, by reducing inflammatory markers and modulating the renin–angiotensin system (RAS).

*Aronia melanocarpa* fruits are extremely rich in anthocyanins, flavonoids, and other polyphenolic compounds, which are the basis of its antioxidant properties. These constituents act as scavengers of free radicals like superoxide anions (O_2_−), hydroxyl radicals (OH−), and hydrogen peroxide (H_2_O_2_), modulating oxidative stress signaling pathways and preventing cellular oxidative damage. These compounds also increase the activity of endogenous antioxidant enzymes such as superoxide dismutase (SOD), catalase (CAT), and glutathione peroxidase (GPx), which play critical roles in the detoxification of reactive oxygen species [53,54]. Aronia has been shown to have anti-inflammatory properties associated with polyphenolic compounds, particularly anthocyanins. The mechanisms of action include inhibition of the nuclear factor kappa-light-chain-enhancer of activated B cells (NF-κB), thereby reducing the expression of pro-inflammatory cytokines such as TNF-α, IL-6, and IL-1β. The anthocyanins in chokeberry juice also inhibit the expression of cyclooxygenase-2 (COX-2) and inducible nitric oxide synthase (iNOS), both of which are involved in the inflammatory cascade. The anti-inflammatory effect is also due to reduced levels of pro-inflammatory mediators such as prostaglandins and cytokines [53,54].

In previous studies, the anti-inflammatory effects of chokeberry, expressed as a reduction in inflammatory markers such as C-reactive protein (CRP), IL-6, and VCAM-1, were observed in post-myocardial infarction patients taking chokeberry extract. These systemic effects reduce inflammation and may modulate the expression of ACE2 and MAS1 [52]. On the other hand, aronia has been found to act as an ACE inhibitor, which could lead to increased expression of ACE2 to compensate for reduced ACE activity. This effect is systemic and involves multiple organs, not just the heart [52].

Aronia has antioxidant and angioprotective properties, which are expressed in the stimulation of nitric oxide (NO) synthesis and has a vasoprotective effect, suggesting a systemic improvement in endothelial function. This improved function may increase ACE2 expression as part of a compensatory mechanism to counter oxidative stress and inflammation [55,56].

Studies in applied models of hypertension and atherosclerosis have shown that aronia consumption leads to a reduction in ACE activity, which correlates with a systemic reduction in inflammation and improvement in vascular function [57,58].

### Clinical Remarks

Among the great therapeutic challenges in cardiology, coronary artery disease (CAD) stands out, characterized by decreased blood flow to the heart resulting from the atherosclerotic process in coronary vessels. This, in turn, leads to hypoxia, ischemic heart failure, and disturbances in rhythm and conduction. The basic principle of improving blood flow is vascular reconstruction by medical vasodilation, stent implantation, or aorto-coronary bypass [59]. The administration of new pharmaceuticals and the implantation of advanced generations of stents demonstrate favorable outcomes in individuals diagnosed with coronary artery disease (CAD). These approaches may prove ineffective in certain individuals with CAD due to factors such as diffuse coronary stenosis, postoperative restenosis, and heart failure after acute myocardial infarction. In the pursuit of new avenues for neo angiogenesis, the current exploration of vascular endothelial-growth-factor-based therapy is fraught with unreliability due to its potential to accelerate angiogenesis within the atherosclerotic plaque, thereby engendering plaque instability. A promising new method for neo angiogenesis is stem-cell-based therapy [60]. In light of these considerations, the concept of therapeutic angiogenesis has gained significant relevance over the past decade. A growing number of studies have appeared in the literature demonstrating that neovascularization can effectively improve blood flow to the ischemic myocardium. There are two main mechanisms by which neovascularization occurs: vasculogenesis and angiogenesis. In the former, the organization of EPCs in capillaries occurs in situ; in the latter, new vessels are formed from existing ones by division and folding [59,60].

The preclinical and clinical aspects in the application of CD34+ cells as an effective therapeutic agent in vascular, ischemic, and inflammatory pathological processes are extensively reviewed by Hassanpour et al. [17]. CD34+ cells are used as a therapeutic agent because of their ability to stimulate angiogenesis, anti-inflammatory effects, and tissue regeneration. They migrate to areas of ischemia or inflammation in response to factors such as SDF-1 and VEGF, promoting vascular repair and preventing tissue damage [40,61].

Clinical applications include improvement of microcirculation in the epicardium and coronary arteries and attenuation of inflammatory responses through secretion of IL-10 and inhibition of NF-κB [41], as well as improving neurological function and angiogenesis in cerebrovascular disease. CD34+ cell therapy has already shown promising results in the treatment of ischemic and inflammatory diseases, especially cardiovascular and cerebrovascular pathologies [61,62,63].

Therapeutically, ACE inhibition is one of the most preferred therapeutic targets to achieve organ protection in the long term. An innovative therapeutic line is the stimulation of ACE 2 production. Several ACE2 activators and Ang 1–7/MasR agonists have been developed. In addition, novel approaches, including oral ACE2 and Ang 1–7 bioencapsulated in plant cells, have been designed and used in preclinical studies, showing promising cardioprotective effects [51]. A highly compelling approach that can complement existing strategies for enhancing blood flow is the incorporation of functional foods into the daily diet of patients with CAD. This represents a convenient, cost-effective, and innocuous method for managing the atherogenic process [39]. The polyphenolic compounds, specifically anthocyanins, present in black chokeberry have demonstrated efficacy in delaying age-related degenerative changes in the heart through multiple mechanisms. These include a reduction in oxidative stress, the attenuation of inflammation, endothelial function improvement, modulation of hyperlipidemia and hypercholesterolemia, lowering of blood pressure, and influencing coagulation processes [39].

## 5. Conclusions

Our results demonstrate that the supplementation of an antioxidant-rich functional beverage derived from Aronia melanocarpa fruit increases both the activity of the ACE2/MAS1 axis and promotes neovascularization within the myocardium. The investigation of these dual effects is a focal point of extensive research aimed at uncovering novel therapeutic approaches for cardiovascular diseases, which currently stand as the predominant cause of mortality in contemporary society. Attaining preventive and therapeutic effects through functional foods/beverages represents a promising natural alternative and an opportunity for promoting healthy aging. The present study is constrained by limitations associated with the animal experimental model and the examination of a restricted number of morphological markers of neovascularization. However, these preliminary findings provide encouragement for the extension and deepening of research in this area.

## Figures and Tables

**Table 1:** Chemical composition of A. melanocarpa fruit juice (taken from Daskalova et al. [24]).

Sugars, g/L						
Fructose		Glucose		Sorbitol		Sucrose
35.8		28.0		105.8		1.1
Organic acids, g/L						
Quinic acid	Malic acid	Ascorbic acid		Citric acid	Oxalic acid	Tartaric acid
3.25	2.39	0.78		0.37	0.019	0.024
Anthocyanins, mg/L						
Cyanidin-3-galactoside		Cyanidin-3-glucoside		Cyanidin-3-arabinoside		Cyanidin-3-xyloside
1498.4		120.1		502.0		4.6
Phenolic compounds, mg/L						
Chlorogenic acid	Neochlorogenic acid	Epicatechin	Rutin	Quercetin-3-β-glucoside	Quercetin	Total polyphenols
1375.6	1543.1	408.2	446.5	228.9	49.6	11,237.4

**Table 2:** Immunoexpression intensity for α-SMA and CD34 in rat myocardium. Semiquantitative analysis.

Myocardium of Left Ventricle	CY	CO	AMJ
α-SMA immunoexpression intensity	+++	+	++
CD34 immunoexpression intensity	+++	+	+++

Legend: −, absent; +, weak; ++, moderate; +++, strong expression.

**Table 3:** Comparison of vessel and capillary density in rat myocardium based on the expression of α-SMA and CD34. Semiquantitative analysis.

Myocardium of Left Ventricle of Rat Heart	CY	CO	AMJ
vessel density	+++	+	++
microvessel density	+++	+	+++

Legend: −, absent; +, weak; ++, moderate; +++, strong expression.

## Data Availability

The original contributions presented in this study are included in the article. Further inquiries can be directed to the corresponding authors.

## Abbreviations

CY, young controls; CO, old controls; AMJ group, animals supplemented with *Aronia melanocarpa* juice; EPCs, endothelial progenitor cells; ACE, angiotensin converting enzyme; ACE2, angiotensin converting enzyme 2, RAAS, renin–angiotensin–aldosteron system; Ang II, angiotensin II; MAS 1, MAS proto oncogene receptor; AT1, type 1 angiotensin II receptor; Ang(1-7), angiotensin 1-7; SDF, stromal-derived factor 1; VEGF, vascular endothelial growth factor; CAD, coronary artery disease; NO, nitric oxide; ROS, reactive oxygen species.
